# Penicillin binding protein 3 of *Staphylococcus aureus* NCTC 8325-4 binds and activates human plasminogen

**DOI:** 10.1186/s13104-016-2190-4

**Published:** 2016-08-04

**Authors:** Riikka Kylväjä, Tuomas Ojalehto, Veera Kainulainen, Ritva Virkola, Benita Westerlund-Wikström

**Affiliations:** 1 General Microbiology, Department of Biosciences, University of Helsinki, P.O.Box 56, FI-00014 University of Helsinki, Helsinki Finland; 2 Thermo Fisher Scientific, Ratastie 2, 01620 Vantaa, Finland; 3 Orion Diagnostica, Koivu-Mankkaan tie 6, 02200 Espoo, Finland; 4 Pharmacology, Faculty of Medicine, University of Helsinki, P.O.Box 63, FI-00014 University of Helsinki, Helsinki Finland

**Keywords:** *Staphylococcus aureus*, Penicillin binding protein, Plasminogen, Plasmin, Adhesion

## Abstract

**Background:**

*Staphylococcus aureus* is a versatile pathogen expressing a number of virulence-associated adhesive molecules. In a previous study, we generated in a secretion-competent *Escherichia coli* strain a library of random FLAG-tag positive (FTP) polypeptides of *S. aureus*. To identify adhesive proteins and gain additional knowledge on putative virulence factors of *S. aureus*, we here screened the FTP library against human serum proteins.

**Findings:**

*Staphylococcus aureus* NCTC 8325-4, origin of the FTP library, adhered to immobilized plasminogen in vitro. In an enzyme-linked immunoassay a C-terminal part of penicillin binding protein 3 (PBP3), included in the FTP library, bound to immobilized plasminogen. We expressed and purified full-length PBP3 and its C-terminal fragments as recombinant proteins. In a time-resolved fluorometry—based assay the PBP3 polypeptides bound to immobilized plasminogen. The polypeptides enhanced formation of plasmin from plasminogen as analyzed by cleavage of a chromogenic plasmin substrate.

**Conclusions:**

The present findings, although preliminary, demonstrate reliably that *S. aureus* NCTC 8325-4 adheres to immobilized plasminogen in vitro and that the adhesion may be mediated by a C-terminal fragment of the PBP3 protein. The full length PBP3 and the penicillin binding C-terminal domain of PBP3 expressed as recombinant proteins bound plasminogen and activated plasminogen to plasmin. These phenomena were inhibited by the lysine analogue ε-aminocaproic acid suggesting that the binding is mediated by lysine residues. A detailed molecular description of surface molecules enhancing the virulence of *S. aureus* will aid in understanding of its pathogenicity and help in design of antibacterial drugs in the future.

**Electronic supplementary material:**

The online version of this article (doi:10.1186/s13104-016-2190-4) contains supplementary material, which is available to authorized users.

## Findings

### Research background

The human plasminogen (Plg)/plasmin system is a key player in the tightly controlled blood fibrinolytic pathway that results in dissolving small, developing blood clots by rapid, non-specific, proteolytic events [1, 2]. Plg circulates in blood as a proenzyme in two inactive forms: Glu-Plg and Lys-Plg, which are activated to the serine protease plasmin by two physiological Plg activators, tissue-type Plg activator (tPA) and urokinase Plg activator (uPA) [3–5]. The conversion of free Plg to plasmin by soluble activators in plasma is inefficient and fibrinolysis is generally initiated as the fibrin network in blood clots binds Plg that is then converted by tPA into active plasmin [2]. uPA binds to uPA receptors on target cells where it activates plasminogen and the event leads to degradation of extracellular matrix and regulation of cell migration, adhesion and proliferation [5, 6].

Many bacterial species can take advantage of the host’s fibrinolytic system when they disseminate within the host and several bacterial species are known for their ability to generate a proteolytic surface from the human Plg/plasmin system. This enables bacteria to break out from blood clots and e.g. degrade components of the extracellular matrix, complement system or tissues of the host [7–10]. These bacterial species express bacterial Plg activators and Plg receptors by which soluble Plg is immobilized onto the bacterial cell surface. Examples of bacterial Plg activators and receptors are the Pla protein of *Yersinia pestis,* streptokinase of streptococci as well as glyceraldehyde 3-phosphate dehydrogenase and enolase of *Lactobacillus* and streptococci [11–17].

*Staphylococcus aureus* colonizes the human nasopharynx often without adverse effects [18, 19]. However, this opportunistic pathogen is also the infectious agent in e.g. boils and furuncles and in more severe diseases such as bacteremia or endocarditis [20]. For preventive measures and treatment of *S. aureus* infections, it is important to identify the molecular mechanisms underlying colonization and state of infection in human beings. Similarly to some other pathogenic bacteria, *S. aureus* can generate a proteolytic surface that assists in invasive infections and has been shown to express the Plg activator staphylokinase as well as various receptors for Plg on its surface [13, 21, 22]. In this report, our aim was to analyze by an alternative approach, whether there are unknown proteins of *S. aureus* that bind and activate Plg and thereby increase its arsenal of virulence factors.

### Methods

#### Bacterial strains and growth conditions

The *​Escherichia coli* -based library secreting random-length FLAG-tag positive (FTP) polypeptides of *S. aureus* as well as the nucleotide sequences of each *S. aureus* gene fragment in the library were available from previous work in our laboratory [23]. Briefly, the library consists of randomly fragmented chromosomal DNA of *S. aureus* subsp. *aureus* NCTC 8325-4 (from now on *S. aureus* NCTC 8325-4). The fragments are mainly 250–1000 bp in length, distributed randomly and evenly on the *S. aureus* NCTC 8325-4 chromosome. The library was generated in our expression vector, which facilitates secretion of foreign FLAG-tagged polypeptides into the growth medium of *E. coli* MKS12 [23]. The library, which covers approximately 32 % of the staphylococcal proteome, includes 1663 clones that cover 950 individual gene sequences expressing FTP polypeptides of *S. aureus* secreted into the *E. coli* MKS12 growth medium. Most of the sequences did not contain a known secretion signal. The folding state of the FTP polypeptides in the growth medium has not been analyzed. The clones of the *E. coli* MKS12 library were cultured in 300 μl Luria-Bertani broth (LB) supplied with ampicillin (150 μg/ml) on 96-well polystyrene plates statically overnight at 37 °C or statically in 3 ml N-minimal medium as described by Majander et al. [24]. Prior to protein analysis, the growth medium was clarified by centrifugation twice as described by Kylväjä et al. [23]. *Enterococcus faecium* ATCC 19434, *Lactobacillus crispatus* ST1 and *S. aureus* NCTC 8325-4 were available from previous work [23, 25] (Ritva Virkola personal communications). *E. coli* BL21 (AI) Δ*slyD* (F–*ompTgal dcm lon hsdSB*(rB- mB-) *araB*::T7RNAP-*tetA* Δ*slyD*::*cat*) originally provided by Michael S. Donnenberg (University of Maryland) was available from previous work [26, 27]. *E. coli* BL21 (DE3) was from Novagen. *E. faecium* ATCC 19434 and *S. aureus* NCTC 8325-4 were grown shaking in 10 ml Todd-Hewitt broth (Difco; BD Biosciences) for 18 h at 37 °C. *L. crispatus* ST1 was cultured statically in 10 ml de Man, Rogosa and Sharpe broth (MRS; Difco) for 18 h at 37 °C. *E. coli* BL21 (AI) Δ*slyD* (pREP4) and BL21 (DE3) were grown in LB broth or on LB agar plates overnight at 37 °C with appropriate antibiotics. The repressor plasmid pREP4 was used for repression of expression from the pQE-type of vectors (Qiagen). To purify the His-PBP3 recombinant proteins, the hosts 1) *E. coli* BL21 (AI) (pREP4) Δ*slyD* harboring the expression plasmid pQE30/PBP3-F, and 2) BL21 (DE3) (pREP4) harboring pET45b/PBP3-C and pET45b/PBP3-C_S634_ were grown shaking overnight at 37 °C in LB broth with antibiotics (Km 25 µg/ml and Amp 150 µg/ml) and diluted 1/20 in LB broth without ampicillin, to avoid binding of ampicillin to the expressed PBP3 derivatives. Then, (1) *E. coli* BL21 (AI) Δ*slyD* (pQE30/PBP3-F) was grown for 2 h at 37 °C and induced with 1 mM IPTG for 2 h at 4 °C, whereas (2) BL21 (DE3) (pET45b/PBP3-C) and BL21 (DE3) (pET45b/PBP3-C_S634_) were grown for 6 h at 37 °C before induction with 2 mM IPTG for 1 h at 37 °C. The cells used in recombinant protein expression were washed once with PBS, pelleted and stored at −20 °C.

#### Adhesion of bacterial cells to immobilized Plg

Human Glu-plasminogen (Glu-Plg; American Diagnostica) and bovine serum albumin (BSA; Sigma-Aldrich) were immobilized on Diagnostic StarFrost slides (Waldemar Knittel, Braunschweig) by applying 40 µl Glu-Plg (20 µg/ml) and 40 µl BSA (25 µg/ml) in phosphate-buffered saline, pH 7.1 (PBS) onto the slides. The slides were incubated in a moist chamber for 18 h at 20 °C, washed slowly shaking for 3 min in 0.1 % BSA/PBS and blocked statically in 2 % BSA/PBS for 2 h at 20 °C. After washing the slides slowly shaking 3 × 5 min in 0.1 % BSA/PBS, 40 µl of bacterial suspensions (5 × 10^8^ bacteria/ml of PBS) were applied onto the slides, which were then incubated statically in a moist chamber for 2 h at 20 °C. The slides were washed slowly shaking 3 × 3 min in 0.1 % BSA/PBS and left to dry for 18 h at 20 °C. Adhered bacteria were visualized by staining of the slides with 0.2 % Loeffler methylene blue (Merck) for 3 min. The slides were washed in tap water, left to dry at 20 °C and the number of adhered bacteria in 20 randomly chosen microscopic fields (of 3.4 × 10^4^ µm^2^) was quantified for each bacterial strain and each target protein using Image-Pro Plus, version 4.0 TM (Media Cybernetics, Inc., USA) and ImageJ 1.47 software (National Institutes of Health, USA) for analysis of bacterial adhesion. The experiment was repeated three times and the result of one representative experiment was used for calculation of average number of adhered bacteria and the standard deviation in 20 randomly chosen microscopic fields.

#### Primary screening of the binding of FTP polypeptides to immobilized Plg

Cleared supernatants of the FTP clones were prepared as described by Kylväjä et al. [23] and 100 μl of each cleared supernatant was analyzed for binding to immobilized Glu-Plg (100 nM) in an enzyme-linked immunoassay (ELISA). Bound FTP peptides were detected with anti-FLAG^®^ M2 monoclonal antibody (0.5 μg/ml in 1 % BSA/PBS; Sigma-Aldrich, RRID:AB_439685) and alkaline phosphatase-conjugated anti-mouse antibody (1 μg/ml in 1 % BSA/PBS), and the absorbance was measured in a Multiscan Titertek recorder (Eflab) at 405 nm as described for other target proteins in Kylväjä et al. [23]. Clarified, cell-free supernatant of MKS12 (pSRP18/0), carrying an empty vector and the clone named ΔArcB, encoding a C-terminal fragment of the ArcB subunit of a multidrug efflux pump (locus tag SAOUHSC 02525), as well as His-tagged enolase of *L. crispatus* ST1 (ST1 Eno) were available from previous work [12, 23] and were used as controls.

#### Construction and purification of His-tagged PBP3 proteins

We constructed the following N-terminally His-tagged PBP3 recombinant proteins: (a) PBP3-C_S634_, which started at the beginning of the transpeptidase domain at residue E327 and was truncated at the C-terminus identically to that of the FTP library polypeptide, (b) a full-length PBP3 polypeptide named PBP3-F, which started from residue D58 and thereby lacked the membrane anchor domain, and (c) the PBP3 C-terminal domain named PBP3-C, which started from residue E327 and covered the penicillin binding transpeptidase domain [28, 29]. To construct the described recombinant proteins, the *pbp*C gene fragment encoding PBP3-F was cloned into the *Sph*I/*Pst*I restriction site of pQE30 (Qiagen) and the gene fragments encoding PBP3-C and PBP3-C_S634_ carrying two additional N-terminal His-residues, to enhance the binding to the affinity matrix used for protein purification, were cloned into the *Pml*I/*Blp*I restriction site in pET45b (Novagen). Primers used were designed on the basis of the *pbpC* sequence of *S. aureus* NCTC 8325 (locus SAOUHSC_01652) as follows: forward of PBP3-F: 5′CCCGCATGCGATGAAAACATTACAGTGAATGAGTCTG; reverse of PBP3-F: 5′GGGCTGCAGTTATTTGTCTTTGTCTTTATTTTTATCATC; forward of PBP3-C and PBP3-C_S634_: 5′CACCACGAAGTAGAAGCATTATTAGATAAACAAATTAAG; reverse of PBP3-C: 5′GGGCTCAGCTTATTTGTCTTTGTCTTTATTTTTATCATC; reverse of PBP3-C_S634_: 5′GGGGCTCAGCTTAAGAGTTAACTCTTGGCTCTC. Plasmid pQE30/PBP3-F was transformed into *E. coli* BL21 (AI) (pREP4) Δ*slyD* to decrease the contamination of the endogenous SlyD protein of *E. coli* in the affinity purification, and plasmids pET45b/PBP3-C and pET45b/PBP3-C_S634_ into *E. coli* BL21 (DE3) for expression of proteins.

Cells expressing the N-terminally His-tagged PBP3-F were lysed with BugBuster^®^ Master Mix (10 ml BugBuster/cells pelleted from 100 ml culture) (Novagen). Protein present in the soluble cell fraction was purified using nickel-nitrilotriacetic acid (Ni–NTA) metal-affinity chromatography matrix under native conditions according to QIA*express* System (Qiagen) with the following minor modifications. All buffers were supplemented with 10 % glycerol to stabilize the recombinant proteins. In addition, the lysis buffer was supplemented with 15 mM imidazole and the wash buffer with 40 mM imidazole. Cells expressing the N-terminally His-tagged PBP3-C and PBP3-C_S634_ were lysed with QIA*express* native lysis buffer (5 ml/g cells), where after PBP3-C_S634_ and PBP3-C in the non-soluble fractions were solubilized with QIA*express* Urea lysis buffer (10 ml/g pellet). The proteins were purified under denaturing conditions using Ni-NTA agarose according to manufacturer’s instructions. After purification under denaturing conditions, the PBP3-C and PBP3-C_S634_ proteins were refolded by changing the urea-containing elution buffer to PBS containing 10 % glycerol in a PD-10 gel filtration column (GE Healthcare). The purification process was monitored by SDS-PAGE analysis and the concentration of the purified proteins was determined by analyzing the whole band intensity from Coomassie-stained SDS-PAGE gels, with BSA as a standard of known concentrations and using the TINA 2.09c software (Rayest Isotopen Meβgeräte). The secondary structure of the proteins was not assessed.

#### Binding of purified His-tagged PBPs to soluble Plg

The binding of purified His-tagged PBP3 proteins to soluble Plg was analyzed by time-resolved fluorometry developed by Hurmalainen et al. [12] and Kukkonen et al. [30] with minor modifications. Briefly, the concentration of all proteins to be analyzed for binding to Plg was adjusted to 625 nM in PBS/10 % glycerol and the analysis was assessed at a final concentration of 100 nM in PBS (100 µl/well). The His-tagged PBP3 proteins, the positive control protein laminin (Lam; Sigma-Aldrich) [31] and negative control protein ΔNarG (locus tag SAOUHSC 02681) available from previous work [23], were immobilized on polystyrene microtiter plates overnight at 21 °C, the plates were washed and blocked with 2 % BSA/PBS. Glu-Plg (1 µg/well) was added to the wells and the plate was incubated for 4 h at 21 °C. For inhibition studies, Glu-Plg in PBS/0.1 % Tween 20 was incubated with ε-aminocaproic acid (EACA; 10 mM) for 15 min at 21 °C prior to its application onto the immobilized target proteins. The binding of Plg was assessed with parallel samples using polyclonal anti-human Plg IgG (720 ng/well, 2 h, 21 °C; American Diagnostica, RRID: AB_400685) and Eu^3+^-labelled anti-rabbit IgG (80 ng/well, 18 h, 4 °C; PerkinElmer) and measured in a VICTOR^®^ time-resolved fluorometer with DELFIA^®^ enhancement solution (PerkinElmer) using filter settings 340 nm (excitation) and 615 nm (emission). The volume was 100 μl in each step except for the enhancement solution that was used at 250 µl. The experiment was repeated twice with very similar outcomes. The average and standard deviation of two parallel samples were calculated on the basis of the results of one of the experiments.

#### Formation of active plasmin by purified proteins

His-tagged PBP3-F, PBP3-C and PBP3-C_S634_ (100 nM), Lam (100 nM) and ΔNarG (100 nM) in PBS/10 % glycerol were mixed in polystyrene microtiter wells with Glu-Plg (4 µg/well), and the tissue-type Plg activator tPA (2 ng/well; Calbiochem) in PBS. Controls included were (1) wells without Plg, (2) wells without tPA, (3) wells without Plg and tPA, and (4) wells without His-tagged PBP3 proteins. The lacking reaction components were substituted with buffer. For inhibition studies, EACA (0.73 mM) was added to the wells 5 min prior to Glu-Plg addition. The chromogenic plasmin substrate SS-2251 (H–D-Val-Leu-Lys-p-nitroanilinehydrochloride; Kabivitrum) was added (0.45 mM) to all wells prior to measurement of the absorbance in a Multiscan Titertek recorder (Eflab) at 405 nm (time point T_0_). Final volume in each well was 200 μl. The absorbance was measured at intervals of 15–30 min for 4. 5 h and values of T_0_ were subtracted from measurements of the other time points as well as the background level of the activation of substrate SS-2251 in buffer. The experiment with parallel samples was repeated twice; the average and standard deviation of two parallel samples were calculated on the basis of the results of one of the experiments.

### Results and discussion

We have previously generated a library of random FLAG-tag positive polypeptides (FTP library) of strain *S. aureus* NCTC 8325-4 in the secretion-competent *E. coli*K12 derivative called MKS12. This strain can secrete heterologous proteins into the growth medium [23, 24] and the heterologous polypeptides can be analyzed directly in the growth medium of *E. coli* MKS12 for binding to various target molecules as described by Majander et al. [24] and Kylväjä et al. [23]. In this report, we first analyzed the adhesion of *S. aureus* NCTC 8325-4 cells to Plg and then assessed the binding to Plg in vitro by *S. aureus* NCTC 8325-4 polypeptides present in the *E. coli* MKS12—based FTP library.

**Fig. 1:**
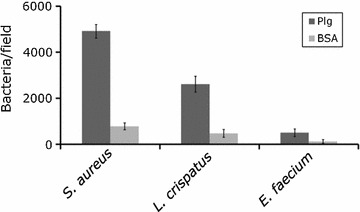
Binding of bacterial cells to immobilized Plg. Glu-Plg (20 μg/ml) and BSA (25 μg/ml) were immobilized onto diagnostic glass slides. *Enterococcus faecium* ATCC 19434, *Lactobacillus crispatus* ST1 and *Staphylococcus aureus* NCTC 8325-4 (5 × 10^8^ cells/ml) were allowed to adhere to the immobilized proteins. Adhered bacteria were visualized by staining with Loeffler’s methylene blue. The number of adhered bacteria was quantified microscopically. The mean and the ± SD of adhered bacteria in 19 or 20 randomly chosen microscopic fields in a representative experiment are shown

Adhesion of *E. faecium* ATCC 19434, *L. crispatus* ST1 and *S. aureus* NCTC 8325-4 to immobilized Plg and BSA was assessed. We chose the strain *S. aureus* NCTC 8325-4 for the adhesion assay, since its chromosome had been used when we constructed the FTP library [23], which also was used for screening purposes in this report. To our knowledge, binding of Plg has been demonstrated with other *S. aureus* strains [21, 22, 32], but never with the commonly used laboratory strain *S. aureus* NCTC 8325-4. *L. crispatus* ST1 has previously been proven to bind and activate Plg and it was used here as a positive control [12, 13]. *E. faecium* has not been demonstrated to interact with Plg and was therefore applied as a putative negative control strain here. In the in vitro adhesion assay, *S. aureus* adhered efficiently to Plg (ca 4830 bacteria per microscopic field), but not to BSA (Fig. 1). The adhesive capacity of *L. crispatus* to Plg was much lower (ca 2580 bacteria per microscopic field), whereas *E. faecium* did not significantly adhere to Plg or BSA (primary data is shown in Additional file 1).

**Fig. 2:**
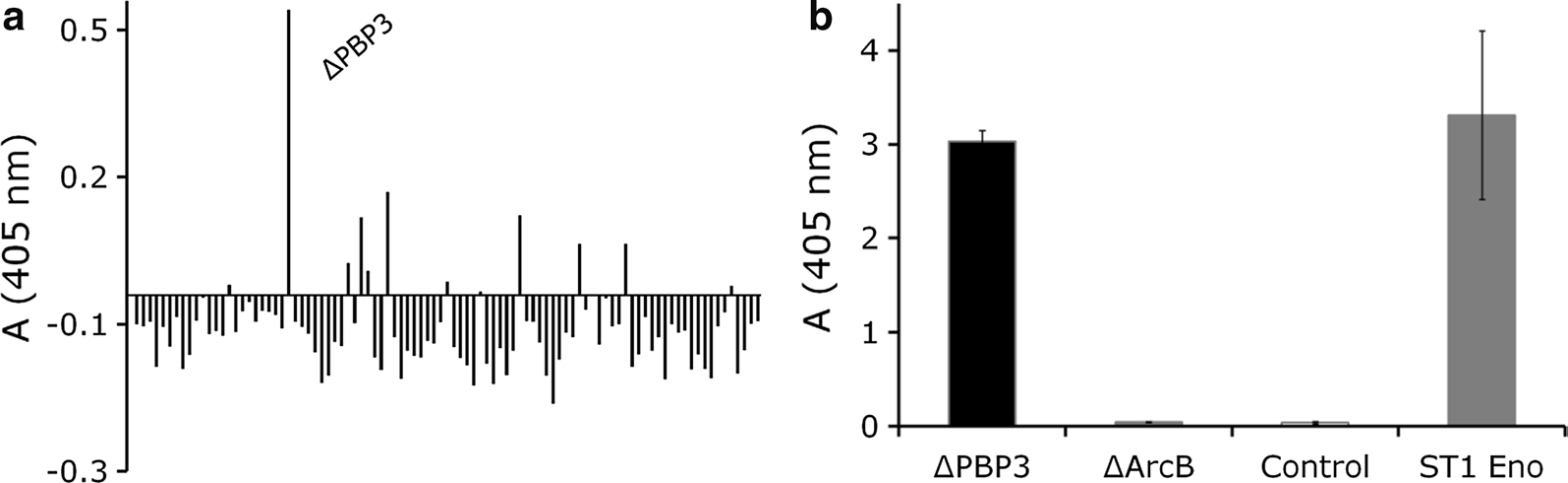
Binding of cell-free growth medium of the FTP library clones to immobilized plasminogen. **a** Binding of clarified growth medium from 95 representative FTP library clones to immobilized plasminogen is shown. The clone encoding a fragment of *S. aureus* PBP3 is named ΔPBP3. Clarified growth medium of the 96th clone, the host strain MKS12 carrying the empty vector pSRP18/0, was used as the background level and the value obtained with this control was subtracted from the other results. The results were measured in ELISA plate reader at A_405nm_. **b** Binding of the clarified growth medium from the ΔPBP3 library clone to immobilized plasminogen is shown. Purified, His-tagged enolase of *Lactobacillus crispatus* ST1 (ST1 Eno) represents a positive control protein and the clarified supernatant from the clone named ΔArcB, encoding a fragment of *S. aureus* ArcB protein, represents the negative control. The binding of clarified supernatant from MKS12 (pSRP18/0), named as Control, is also shown. The results were measured in ELISA plate recorder at A_405nm_. The mean and ± SD of duplicate samples from a representative experiment are shown

**Fig. 3:**
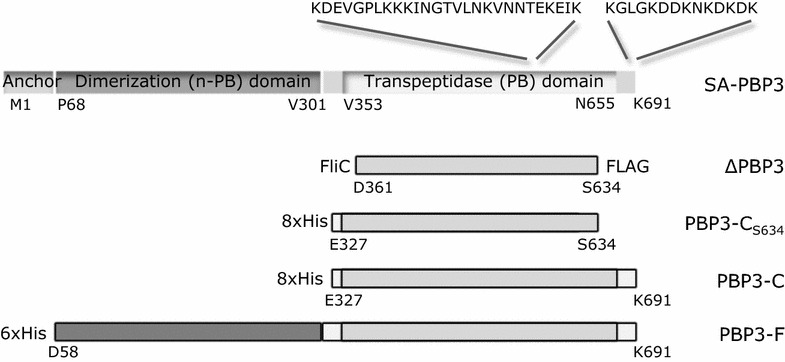
Schematic presentation of *S. aureus* PBP3 and PBP3 fragments expressed as His-tagged recombinant proteins. The PBP3 of *S. aureus* (SA-PBP3) with a non-penicillin binding (n-PB) dimerization domain connected by an inter-domain hinge to a penicillin binding (PB) transpeptidase domain is shown uppermost. ΔPBP3 is encoded by the FTP library clone and it carries an N-terminal signal for secretion (FliC) and a C-terminal FLAG-tag encoded by the expression vector pSRP18/0. The recombinant protein named PBP3-C_S634_ carries eight N-terminal histidine residues and the truncated sequence of the penicillin binding (PB) domain of PBP3. The recombinant protein named PBP3-C carries eight N-terminal histidine residues and the sequence of the penicillin binding (PB) domain of PBP3. The recombinant protein PBP3-F carries an N-terminal hexahistidine tag (His), the non-penicillin (n-PB) and penicillin binding (PB) domains, but lacks the N-terminal membrane anchor sequence (Anchor) of native PBP3. The N- and C-terminal amino acid residues of the domains are indicated below each protein. The lysine-rich sequences of SA-PBP3 are indicated (KDEVGPLKKKINGTVLNKVNNTEKEIK and KGLGKDDKNKDKDK)

*Staphylococcus aureus* is known to express the single plasminogen activator staphylokinase and a few plasminogen receptors, such as Sbi, Ebh and α-enolase, on its surface [13, 22, 32, 33]. We next wanted to examine whether *S. aureus* NCTC 8325-4 expresses putative Plg receptors in addition to the well-known ones. To explore that, we screened the polypeptides of the FTP library for their binding to Plg (Fig. 2a, primary data is shown in Additional file 2). One specific library clone, named ΔPBP3, attracted our attention. The extracellular polypeptide secreted by the ΔPBP3 clone bound to Plg in an ELISA assay much more efficiently than peptides secreted by any other clone of the FTP library (Fig. 2a). We next confirmed the result obtained from the screening experiment with the Plg-binding clone ΔPBP3 (Fig. 2b). The clarified growth medium from the ΔPBP3 library clone bound to immobilized plasminogen as well as the enolase of *L. crispatus* ST1 (ST1 Eno), reported to bind plasminogen [12, 13]. The clarified growth medium of the clone named ΔArcB or the host strain MKS12 (pSRP18/0) used as negative controls did not bind to immobilized Plg (Fig. 2b, primary data is shown in Additional file 2). Based on the sequence of the *S. aureus* gene carried by clone ΔPBP3, the gene product and thereby the polypeptide secreted into the *E. coli* growth medium is a C-terminal fragment of penicillin binding protein 3 (PBP3; locus tag SAOUHSC 02430) (Fig. 3) [23].

Penicillin binding proteins (PBPs) are involved in the last steps of peptidoglycan biosynthesis in bacteria [34]. The penicillin binding domains of PBPs are targets of β-lactam antibiotics including penicillin, hence the name [28, 35]. The β-lactam antibiotics mimic the PBP substrate and bind irreversibly to a conserved serine residue in the active groove of the enzyme, thereby inhibiting its activity [36, 37]. PBPs are multimodular proteins that are classified into high-molecular-weight (HMW) and low-molecular-weight (LMW) PBPs. HMW PBPs are further classified as class A or class B PBPs and the LMW PBPs that function as carboxypeptidases are also referred to as class C PBSs [35]. The HMW PBPs of class A are bifunctional enzymes containing (1) a non-penicillin binding glycosyltransferase (GTase) domain that catalyzes elongation of the glycan chain of peptidoglycan and (2) a C-terminal, penicillin binding transpeptidase (TPase) domain that crosslinks the peptidoglycan. The only known enzymatic function for class B HMW PBPs is the TPase activity of the C-terminal penicillin binding domain (Fig. 3). The N-terminal dimerization domain of class B PBPs has been postulated to act as an intramolecular chaperone that assists the proper folding of the C-terminal domain or have a role in cell morphogenesis [28, 38].

PBP3 is one of the four penicillin binding proteins (named PBP1-PBP4) of methicillin sensitive *S. aureus* and belongs to class B HMW PBPs [39–42]. PBP3 is considered as an enzyme non-essential for growth and survival of *S. aureus* [42]. The specific role for PBP3 is not known. The cell morphology of mutant strains lacking the *pbpC* gene that encodes PBP3 do not differ significantly from cells expressing PBP3, but the mutant cells exhibit increased methicillin resistance [29, 42].

**Fig. 4:**
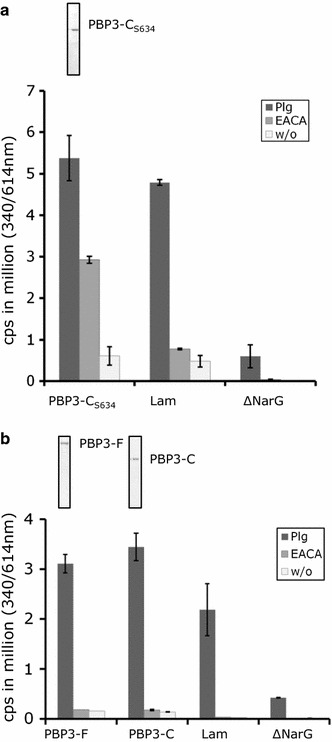
Binding of soluble plasminogen to His-tagged recombinant PBP3 proteins. The binding of soluble plasminogen to immobilized **a** PBP3-C_S634_ and **b** PBP3-F and PBP3-C was measured with VICTOR^®^ time-resolved fluorometer as counts per second (cps) at 340/615 nm. Laminin (Lam) and ΔNarG were included as controls. The test included binding in the presence of the proteins to be tested and (i) Plg (*black bars*, Plg), (ii) Plg and EACA (*grey bars*, EACA), (iii) binding in the absence of Plg or EACA (*white bars*, w/o). The mean and the ±SD of duplicate samples of one representative experiment are shown. SDS-PAGE analysis of the purified PBP3-C_S634_, PBP3-F and PBP3-C recombinant proteins are shown above the respective fluorometry results in figure **a** and **b**

To verify the Plg binding properties of PBP3 that we observed with the ΔPBP3 FTP polypeptide, we cloned and expressed an N-terminally His-tagged polypeptide named PBP3-C_S634_, which started at the beginning of the transpeptidase domain at residue E327, and was truncated at the C-terminus identically to the FTP library polypeptide (Fig. 3). SDS-PAGE analysis of the purified PBP3-C_S634_ polypeptide is shown in Fig. 4a. The binding of PBP3-C_S634_ to human Glu-Plg was assessed by time-resolved fluorometry, where the His-tagged PBP3-C_S634_ protein was immobilized in polystyrene wells and soluble Plg was allowed to bind. This setup mimics the situation in vivo, where Plg receptors are attached to the bacterial surface and soluble Plg is recruited to the surface by the Plg receptors. Immobilization of Plg/plasmin onto the bacterial surface, or onto host tissues, enhances the plasmin formation and protects the activated plasmin from physiological inhibitors such as α2-antiplasmin [1, 4]. PBP3-C_S634_, as well as the control protein laminin, bound efficiently Plg, whereas ΔNarG, which was used as a negative control protein, did not bind Plg (Fig. 4a, Primary data is shown in Additional file 3). C–terminally located and internal repetitive lysine residues, as well as other positively charged amino acid motifs, present in various Plg-binding proteins are known to bind to the kringle domains of Plg. The lysine-mediated binding can be inhibited by the lysine analogue EACA [43–48]. The binding of immobilized PBP3-C_S634_ to soluble Plg was inhibited by EACA, which indicates that lysine residues are involved in the binding (Fig. 4a). PBP3 contains lysine residues at the ultimate C-terminus (Fig. 3, SA-PBP3), but the ΔPBP3 FTP polypeptide and the PBP3-C_S634_ did not include the C-terminal lysine-rich sequence of PBP3 (Fig. 3, ΔPBP3 and PBP3-C_S634_). The results obtained with truncated, recombinant PBP3-C polypeptides suggest that other residues than C-terminal lysine residues are important in the binding of soluble Plg to PBP3 of *S. aureus*. This hypothesis is supported by the fact that ΔPBP3 and PBP3-C_S634 _ contain an internal lysine-rich region (KDEVGPLKKKINGTVLNKVNNTEKEIK) located 104 amino acid residues N-terminally from the ultimate C-terminus of PBP3 (Fig. 3). Enolase of streptococci has similarly been shown to bind and activate Plg via internal, surface-exposed lysine residues [49].

We next wanted to test the binding of the full length PBP3 and the full C-terminal transpeptidase domain of the PBP3 protein to soluble Plg similarly as we did with the PBP3-C_S634_ protein. The genes encoding the His-tagged PBP3 polypeptide named PBP3-F, which started from residue D58 and thereby lacked the membrane anchor domain, and the His-tagged C-terminal domain named PBP3-C, which started from residue E327 and covered the penicillin binding transpeptidase domain of the protein [28, 29] (Fig. 3), were cloned and the corresponding gene products were purified. SDS-PAGE analysis of purified PBP3-F and PBP3-C is shown in Fig. 4b. PBP3-F and PBP3-C both bound soluble Plg similarly as the PBP3-C_S634_ protein and EACA inhibited the binding (Fig. 4b, Primary data is shown in Additional file 3).

**Fig. 5:**
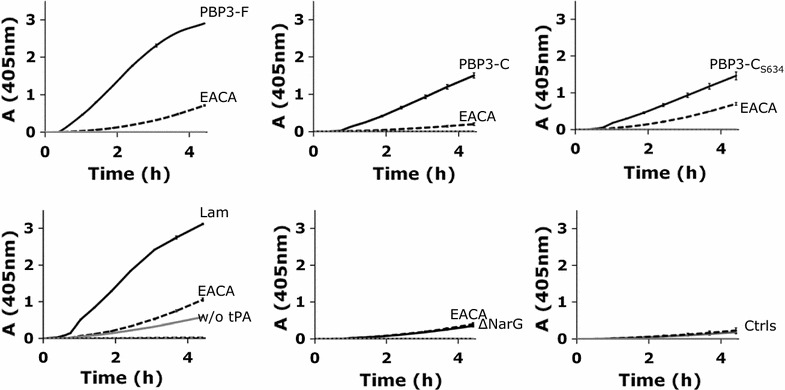
Enhancement of plasmin formation by His-tagged recombinant PBP3 proteins. The activation of plasminogen to plasmin by PBP3-F, PBP3-C, PBP3-C_S634_, Lam and ΔNarG was measured in ELISA plate recorder (A_405nm_) at 15–30 min time intervals for 4.5 h. The background level with buffer only (Bfr) is also shown. The test included plasminogen activation by the corresponding protein (PBP3-F, PBP3-C, PBP3-C_S634_, Lam, ΔNarG) or buffer only (Ctrls) in the presence of (i) Plg, tPA and the substrate, (ii) Plg, tPA, EACA and the substrate (EACA), (iii) Plg and the substrate without tPA (w/o tPA), iv) tPA and the substrate without Plg and (v) substrate in buffer. The mean and ±SD of the duplicate samples from one representative experiment are shown

When soluble Plg is immobilized by Plg receptors on the bacterial surface in vivo, it is converted to active plasmin by the physiological activators tPA or uPA [10] or by bacterial activators [32]. We next assessed whether tPA activated the PBP3-bound, proteolytically inactive Plg into active plasmin. Our results, obtained by measuring the cleavage of a chromogenic plasmin substrate, showed that plasminogen was activated into plasmin in the presence of PBP3-F, PBP3-C or PBP3-C_S634_ and tPA (Fig. 5). The positive control protein laminin also enhanced tPA-mediated activation of Plg into plasmin whereas ΔNarG had no effect on plasmin formation. PBP3-F, PBP3-C, PBP3-C_S634_ or laminin did not activate Plg directly into plasmin when tPA was not present (Fig. 5, w/o tPA), indicating that PBP3 does not function as a direct Plg activator. The tPA mediated plasmin activity was not enhanced in the presence of EACA implying that lysine residues are important in the interaction of Plg and PBP3 proteins (Fig. 5, EACA). The binding of Plg to the putative Plg receptors PBP3-F, PBP3-C or PBP3-C_S634_ as well as the control protein laminin, was required for tPA mediated activity since Plg and tPA alone did not provoke plasmin activity (Fig. 5, Ctrls). Furthermore, our results show that PBP3-F, PBP3-C and PBP3-C_S634_ did not alone, when tPA and Plg were not present, cleave the chromogenic substrate although the PBP3 proteins contain an active-site serine similarly as the serine proteases that cleave the chromogenic substrates (Fig. 5, w/o tPA or Plg) [29, 50]. Primary data of Fig. 5 is shown in Additional file 4. Altogether these results imply that the PBP3 protein acts as a Plg receptor in vitro. The enhanced plasmin activity is mediated by tPA only after the inactive, soluble Plg is bound by PBP3-F, PBP3-C or PBP3-C_S634_ via internal lysine residues.

The penicillin binding domain of PBP3 that synthesizes the last steps in *S. aureus* cell wall polymerization is located outermost from the cell membrane in PBP3, but it is currently not known whether it is exposed e.g. during the cell wall turnover or released to the extracellular milieu. Surface exposure would enable binding of soluble Plg by this putative Plg receptor. The biological role of PBP3 and other PBPs in plasmin formation in vivo remains still to be revealed. Muthukrishnan et al. [51] have shown that planktonic, nasal carrier strains of *S. aureus* secrete PBPs among other molecules. Additionally, PBP2 of *S. aureus* has been shown to protrude to the extracellular environment in methicillin resistant S*. aureus* [52] and to be localized to membrane vesicles of *S. aureus* ATCC 14458 [53]. Other types of PBPs have been found in the exoproteome of other bacterial species, such as *Clostridium difficile*, *Streptococcus agalactiae* and *Corynebacterium pseudotuberculosis* [54–56]. These findings imply that PBPs might have moonlighting functions [57] outside the bacterial cell wall. In this study, the full length His-tagged PBP3 clearly bound Plg in vitro and enhanced the tPA mediated activation of bound plasminogen. The binding was localized to the C-terminal domain of PBP3, where repetitive internal lysine residues reside. These results imply the putative role of PBP3 as a Plg receptor on the cell surface of *S. aureus.* However, the presence and the role of PBP3, and other PBPs, on cell surface or in the exoproteome of *S. aureus* should be further studied.

Plg activation is a well-known virulence trait of many bacterial species [11] and the activation might lead to invasive infections. *S. aureus* is carried in the nasopharynx by 20–40 % of human population and the carrier status can lead to severe invasive infections [58, 59]. The nasal carrier strains of *S. aureus* have been shown to secrete PBPs [51, 60] and their role in invasive infections should be further studied. The results presented in this study indicate that PBPs might have a moonlighting role as Plg receptors in invasive infections.

## Additional files

10.1186/s13104-016-2190-4 Primary data of bacterial adhesion to Plg and BSA (Fig. 1).
10.1186/s13104-016-2190-4 Primary data of plasminogen binding by FTP library clones (Fig. 2).
10.1186/s13104-016-2190-4 Primary data of Plg binding by recombinant proteins (Fig. 4).
10.1186/s13104-016-2190-4 Primary data of Plg activation by recombinant proteins (Fig. 5).

## Abbreviations

BSA: bovine serum albumin
PBP3-C: C-terminal domain of penicillin binding protein 3
EACA: ε-aminocaproic acid
ELISA: enzyme-linked immunoassay
FTP: FLAG-tag positive
PBP3-F: full length penicillin binding protein 3 without the membrane anchor domain
GTase: glycosyltransferase
HMW: high-molecular-weight
IPTG: isopropyl β-D-1-thiogalactopyranoside
LMW: low-molecular-weight
LB broth: Luria–Bertani broth
MRS broth: de Man, Rogosa and Sharpe broth
n-PB: non-penicillin binding
Ni–NTA: nickel-nitrilotriacetic acid
PB: penicillin binding
PBP: penicillin binding protein
PBS: phosphate-buffered saline
Plg: plasminogen
ST1 Eno: His-tagged enolase of *L. crispatus* ST1
tPA: tissue-type Plg activator
TPase: transpeptidase
uPA: urokinase Plg activator
